# Development of dynamical network biomarkers for regulation in Epstein-Barr virus positive peripheral T cell lymphoma unspecified type

**DOI:** 10.3389/fgene.2022.966247

**Published:** 2022-12-05

**Authors:** Dan Shen, Yin Hong, Zhengyang Feng, Xiangying Chen, Yuxing Cai, Qiliang Peng, Jian Tu

**Affiliations:** ^1^ Department of Oncology, The Third Affiliated Hospital of Chongqing Medical University, Chongqing, China; ^2^ Department of Cardiothoracic Surgery, Suzhou BenQ Hospital, Suzhou, China; ^3^ Department of Oncology, The Second Affiliated Hospital of Soochow University, Suzhou, China; ^4^ Department of Radiotherapy and Oncology, The Second Affiliated Hospital of Soochow University, Suzhou, China; ^5^ Department of Pathology, The Second Affiliated Hospital of Soochow University, Suzhou, China

**Keywords:** peripheral T cell lymphoma unspecified type (PTCL-U), Epstein-Barr virus (EBV), gene regulatory network, bioinformatics analysis, network biomarkers

## Abstract

**Background:** This study was performed to identify key regulatory network biomarkers including transcription factors (TFs), miRNAs and lncRNAs that may affect the oncogenesis of EBV positive PTCL-U.

**Methods:** GSE34143 dataset was downloaded and analyzed to identify differentially expressed genes (DEGs) between EBV positive PTCL-U and normal samples. Gene ontology and pathway enrichment analyses were performed to illustrate the potential function of the DEGs. Then, key regulators including TFs, miRNAs and lncRNAs involved in EBV positive PTCL-U were identified by constructing TF–mRNA, lncRNA–miRNA–mRNA, and EBV encoded miRNA–mRNA regulatory networks.

**Results:** A total of 96 DEGs were identified between EBV positive PTCL-U and normal tissues, which were related to immune responses, B cell receptor signaling pathway, chemokine activity. Pathway analysis indicated that the DEGs were mainly enriched in cytokine-cytokine receptor interaction and chemokine signaling pathway. Based on the TF network, hub TFs were identified regulate the target DEGs. Afterwards, a ceRNA network was constructed, in which miR-181(a/b/c/d) and lncRNA LINC01744 were found. According to the EBV-related miRNA regulatory network, CXCL10 and CXCL11 were found to be regulated by EBV-miR-BART1-3p and EBV-miR-BHRF1-3, respectively. By integrating the three networks, some key regulators were found and may serve as potential network biomarkers in the regulation of EBV positive PTCL-U.

**Conclusion:** The network-based approach of the present study identified potential biomarkers including transcription factors, miRNAs, lncRNAs and EBV-related miRNAs involved in EBV positive PTCL-U, assisting us in understanding the molecular mechanisms that underlie the carcinogenesis and progression of EBV positive PTCL-U.

## Introduction

Peripheral T cell lymphoma, unspecified (PTCL−U) is one of the most common subtypes of mature post-thymic T cell neoplasms, which consists of variable immunophenotypes and morphological changes (Broccoli and Zinzani, 2017). PTCL-U is a rare and aggressive disease of the elderly with a low 5-year survival rate (approximately 16%) (Pileri et al., 2021). PTCL-U is a highly heterogeneous disease and its pathogenesis remains unclear. Epstein-Barr virus (EBV) is a well-characterized oncovirus, associated with several malignancies (Kanda et al., 2019). As we all know, there are three main diseases caused by or associated with EBV infection: extranodal NK/T cell lymphoma nasal type (ENKTL), nasopharyngeal carcinoma and infectious mononucleosis (Farrell, 2019). In addition, EBV has also been associated with the development of multiple other tumors including gastric cancer, colorectal cancer and lymphoma (Vockerodt et al., 2015; Fernandes et al., 2020; Saito and Kono, 2021). EBV is implicated in numerous reactive and neoplastic processes of the immune system (Chen, 2011). EBV can infect T cells, natural killer (NK) cells and other immunocytes and can also be latent in human lymphoid tissue for a long time (Pang et al., 2009). When the body’s immune function is low, latent EB virus activates to form a relapse infection. Nevertheless, the pathogenic mechanism of EBV positive PTCL-U remains elusive.

Gene expression regulation is one of the key mechanisms for controlling biological processes in the organism (Hill et al., 2021). Transcription factors (TFs) are a group of proteins that can bind specifically to the enhancer or promoter DNA regions adjacent to the target genes that they regulate (Bushweller, 2019). microRNAs (miRNAs) are small single-stranded non-coding RNA molecules that can regulate gene expression by forming complementary duplexes with their target mRNAs, leading to translational inhibition and degradation of the target mRNAs (Treiber et al., 2019). It is worth noting that miRNAs are taking increasingly important role in cancer carcinogenesis and their special characteristics made them stable enough to serve as ideal biomarkers and therapeutic target in cancers (Berindan-Neagoe et al., 2014). Long non-coding RNAs (lncRNAs), a diverse family of ncRNAs larger than 200 bp in length without apparent coding potential, play widespread roles in virtually every biological process inplants (Liu et al., 2021a). Moreover, lncRNAs can serve as competing endogenous RNAs (ceRNAs) to sponge their targeted miRNAs and thus influence the biological processes of miRNAs and the downstream mRNAs (Zhong et al., 2018). EBV was the first virus in which viral miRNAs were identified (Forte and Luftig, 2011). EBV-related miRNAs were first detected in Burkitt lymphoma and were found to be associated with the carcinogenesis of lymphomas (De Falco et al., 2009). EBV-encoded miRNAs have been shown to be involved in many biological processes, such as growth and proliferation, immune regulation and the cell cycle (Liu et al., 2021b). These EBV-encoded miRNAs were confirmed with vital roles in promoting viral latency or cancer progression through targeting both viral and cellular genes (Wang et al., 2019a). Further studies are needed to elucidate the functions of most EBV-encoded miRNAs.

Recently, bioinformatics analysis as an interdisciplinary field has been regarded as a reliable technique for revealing molecular markers and therapeutic targets that may be implicated in the pathogenesis of cancer or other diseases (Gendoo, 2020). In the era of big biological data, the application of computational biology to process and analyze data quickly and efficiently may be the key to solve the high cost and long lead time of drug development for rare diseases. Since TFs and ncRNAs are important regulators in cancer progression, construction of gene regulatory networks on the basis of TFs and ncRNAs may help obtain a more comprehensive understanding of biological processes of EBV positive PTCL-U. Although cases of EBV positive PTCL-U are rare, the microarray and bioinformatics analyses have provided a new way to study the molecular mechanism of them.

**FIGURE 1:**
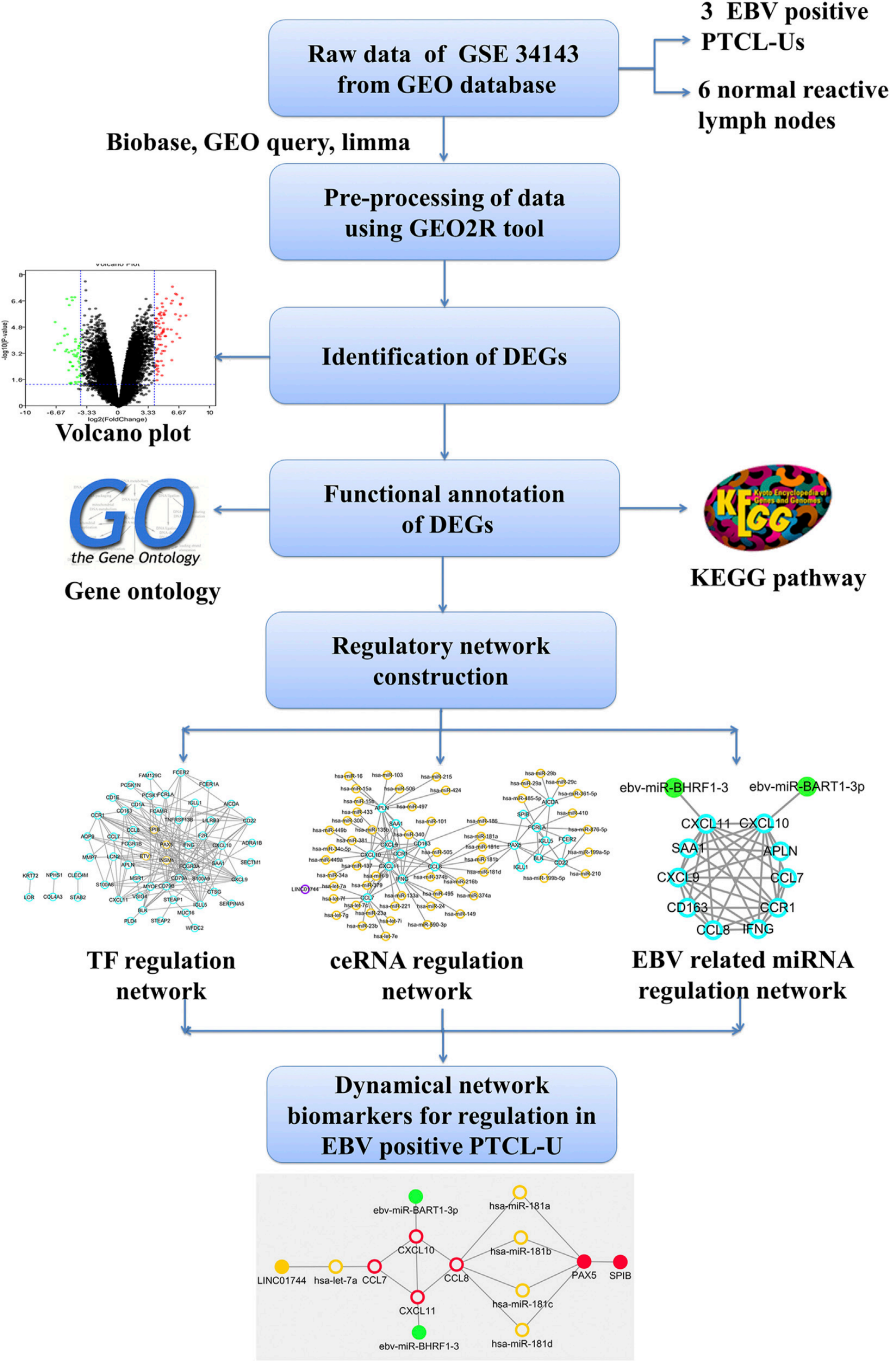
Outline of the workflow and expression data analysis in the present study using bioinformatic strategies.

In this study, some key regulators including TFs, miRNAs and lncRNAs involved in EBV positive PTCL-U were identified and integrated them into a strong theoretical framework through constructing TF–mRNA, lncRNA–miRNA–mRNA, and EBV encoded miRNA–mRNA regulatory networks. The present study may offer new insights into the molecular mechanism of EBV positive PTCL-U and provide potential network biomarkers for EBV positive PTCL-U. The flowchart diagram for this study is represented in Figure 1.

## Materials and methods

### Data collection

The microarray data for GSE34143 in the GEO database were obtained on the GPL6480 and GPL14550 platform. The expression profiles are provided for nine samples, including 3 EBV positive PTCL-U and 6 normal reactive lymph nodes.

### Data processing of differentially expressed genes

GEO2R is an interactive web tool that allows users to compare two or more sets of samples in the GEO Series and contributes to filtrating DEGs through the Bioconductor’s GEO Query and Limma R software (Barrett et al., 2013). We used GEO2R to identify differentially expressed genes (DEGs) between EBV positive PTCL-U and control lymph node tissues. These DEGs were identified as key genes that may play an important part in the occurrence and development of PTCL-U. |LogFC| > 4 and *p*-value < 0.05 were considered statistically significant. The analysis results were presented by volcano map.

### Functional and pathway enrichment analysis

To further evaluate the function of the DEGs, KEGG pathway and GO enrichment analyses were conducted. The GO functional enrichment analysis was applied to annotate genes and gene products, and also accomplish characteristic biological function originating from genomic or transcript omic data at three different levels including biological process (BP), molecular function (MF), and cellular component (CC) (The Gene Ontology, 2017). KEGG has been recognized as an integrated approach containing biological interpretation of genome sequences and other high-throughput data (Kanehisa et al., 2021). In this study, all the key DEGs were mapped to the online tool Database for Annotation, Visualization and Integrated Discovery (DAVID) to perform Gene Ontology (GO) and Kyoto Encyclopedia of Genes and Genomes (KEGG) pathway analyses (Dennis et al., 2003). *p*-value < 0.05 was considered statistically significant.

### Construction of transcription factor regulation network

Search Tool for the Retrieval of Interacting Genes (STRING) is a database resource that can investigate complex PPI information (Szklarczyk et al., 2021). We retrieved the PPI information by mapping DEGs to the STRING database. PPI data with a combined score > 0.4 were chosen. Afterwards, the PPI interaction network was set up and re-analyzed with Cytoscape (Shannon et al., 2003). Moreover, differentially expressed transcription factors (TFs) among the DEGs were identified by querying the Checkpoint database. We built a TFs regulatory network using Cytoscape.

### Construction of competing endogenous RNAs regulatory network

Network analysis was carried out to identify the key hub mRNAs with high degrees in the initial network. Module analysis was applied to screen the significant modules active in the network with the Molecular Complex Detection (MCODE) plugin in Cytoscape (Bader and Hogue, 2003). Then, we conducted a systematic analysis on the interactions between miRNAs and key hub mRNAs using three common databases including TargetScan, microRNA.org and miRDB, based on three different target prediction algorithms (Lewis et al., 2005; Betel et al., 2008; Wong and Wang, 2015). To reduce false positive predictions, the miRNA-mRNA regulatory pairs that identified by the three databases were retrieved. Furthermore, we predicted miRNA-targeted lncRNA interactions by using starBase database resource (Li et al., 2014). A ceRNA interaction network was then constructed by applying Cytoscape software.

### Construction of the Epstein-Barr virus related microRNAs regulatory network

ViRBase (http://www.rna-society.org/virbase) is an online tool that provide a resource for efficient browsing and visualization of interaction networks in virus-host non-coding RNAs (ncRNAs) associated interactions (Li et al., 2015a). In 2021, the ViRBase database has been updated to version 3.0 that provides a more comprehensive resource for virus and host ncRNA-related interactions enabling investigators a more effective methods for studying viral infections (Cheng et al., 2021). Using ViRBase database resource, we predicted the EBV related miRNAs and then established an EBV related miRNA regulatory network with the Cytoscape tool.

### Identification of network biomarkers in peripheral T cell lymphoma unspecified type

The overlapping genes, TFs, miRNAs and lncRNAs in the above three networks were further identified. Next, the cross network among the overlapping genes and regulators were constructed by using Cytoscape software and identified as network biomarkers involved in PTCL-U.

## Results

### Identification of differentially expressed genes in Epstein-Barr virus positive peripheral T cell lymphoma unspecified type

**FIGURE 2:**
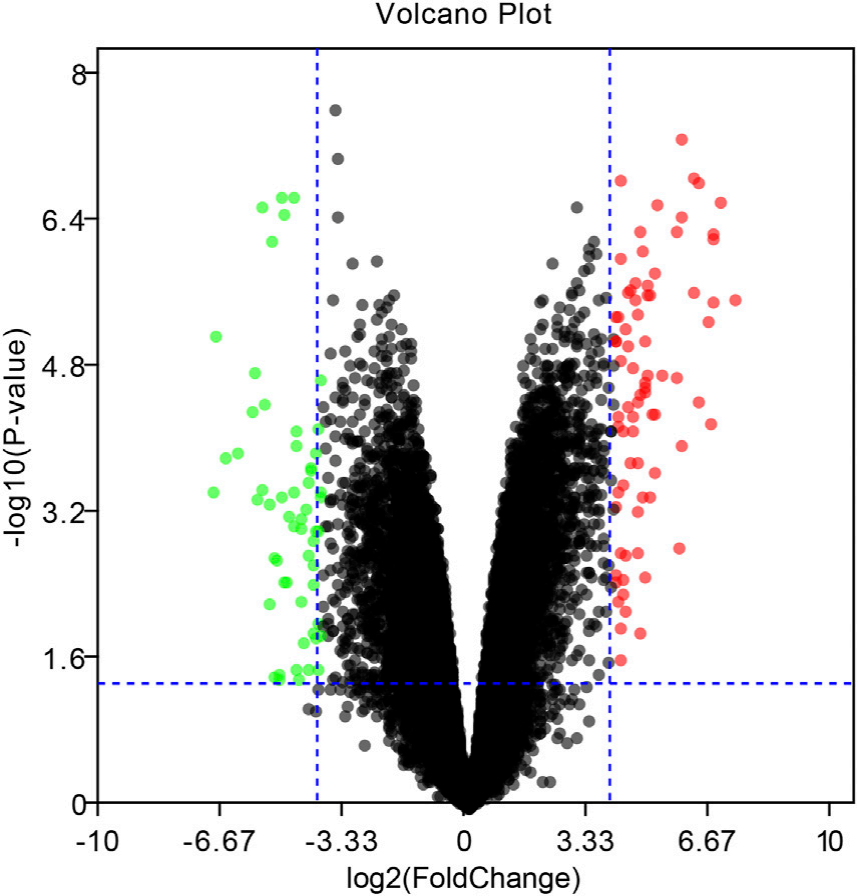
Volcano plot of the differentially expressed genes (DEGs) in EBV positive PTCL-U compared to normal controls in GSE34143. Upregulated DEGs are mapped as red spots, and downregulated DEGs are presented as green spots. Genes without notable variation are labeled as black spots (cutoff values of |logFC| > 4 and *p*-value < 0.05).

To identify DEGs between EBV positive PTCL-U and normal reactive lymph nodes, we retrieved relevant microarray expression profiles of GSE34143 from GEO database. According to the GEO2R analysis with the threshold value, a total of 96 DEGs were identified, among which 52 were upregulated and 44 were downregulated between EBV positive PTCL-U and normal lymph nodes. A volcano plot that presented all DEGs was shown in Figure 2.

### Gene ontology and Kyoto Encyclopedia of Genes and Genomes pathway functional enrichment analyses of differentially expressed genes

**FIGURE 3:**
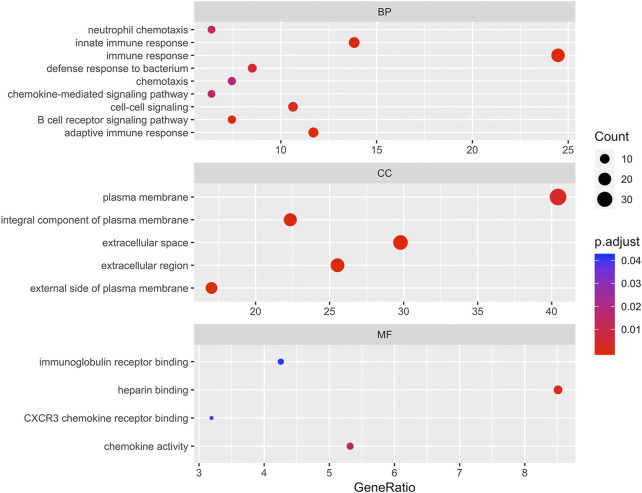
Bubbleplot for gene ontology (GO) enrichment of differentially expressed genes (DEGs). The x-axis label represents gene ratio while y-axis label represents GO terms. The size of the dot represents the number of genes in different functional groups, while the color of the dot reflects the different *p*-value range.

To determine the biological features of DEGs, GO and KEGG pathway analyses, were performed. As shown in Figure 3, the BP analysis revealed that the DEGs were mainly associated with innate immune response, immune response, chemotaxis, chemokine-mediated signaling pathway, cell-cell signaling, B cell receptor signaling pathway, and adaptive immune response. The CC analysis showed that DEGs were enriched in plasma membrane, integral component of plasma membrane, extracellular space, extracellular region, and external side of plasma membrane. Changes in MF of DEGs were significantly enriched in immunoglobulin receptor binding, heparin binding, CXCR3 chemokine receptor binding, and chemokine activity.

**FIGURE 4:**
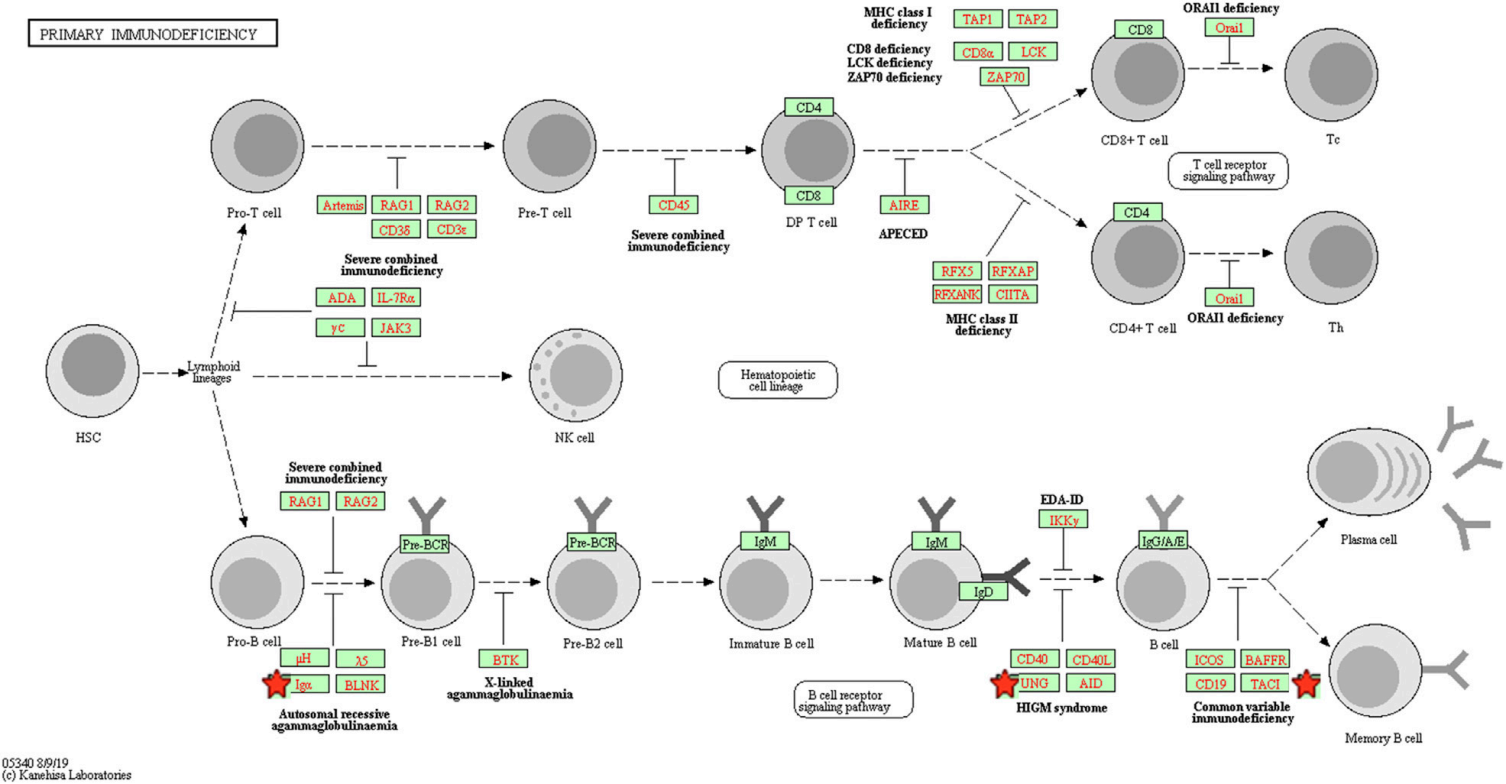
The primary immunodeficiency signaling pathway enriched in KEGG. Objects with pentagrams are acting locus by mapped genes. KEGG, Kyoto encyclopedia of genes and genomes.

To explore the potential mechanism of these DEGs, KEGG pathway analysis was performed using DAVID online tools. By KEGG pathway analysis, the DEGs were mainly associated with cytokine-cytokine receptor interactions, chemokine signaling pathways, hematopoietic cell lineage, primary immunodeficiency and intestinal immune network for IgA production (Table 1). Since these DEGs were highly involved in immune response, we selected the enriched primary immunodeficiency signaling as a significant example, which was also closely connected with B cell receptor signaling pathway, T cell receptor signaling pathway, and hematopoietic cell lineage (Figure 4).

**TABLE 1:** The significantly enriched KEGG pathways of the differentially expressed genes (DEGs).

No.	Pathway term	Number of enriched genes	*p*-value
1	Cytokine-cytokine receptor interaction	8	3.11E-04
2	Chemokine signaling pathway	6	3.33E-03
3	Hematopoietic cell lineage	4	0.012
4	Primary immunodeficiency	3	0.015
5	Intestinal immune network for IgA production	3	0.027

### Transcription factor regulation network

**FIGURE 5:**
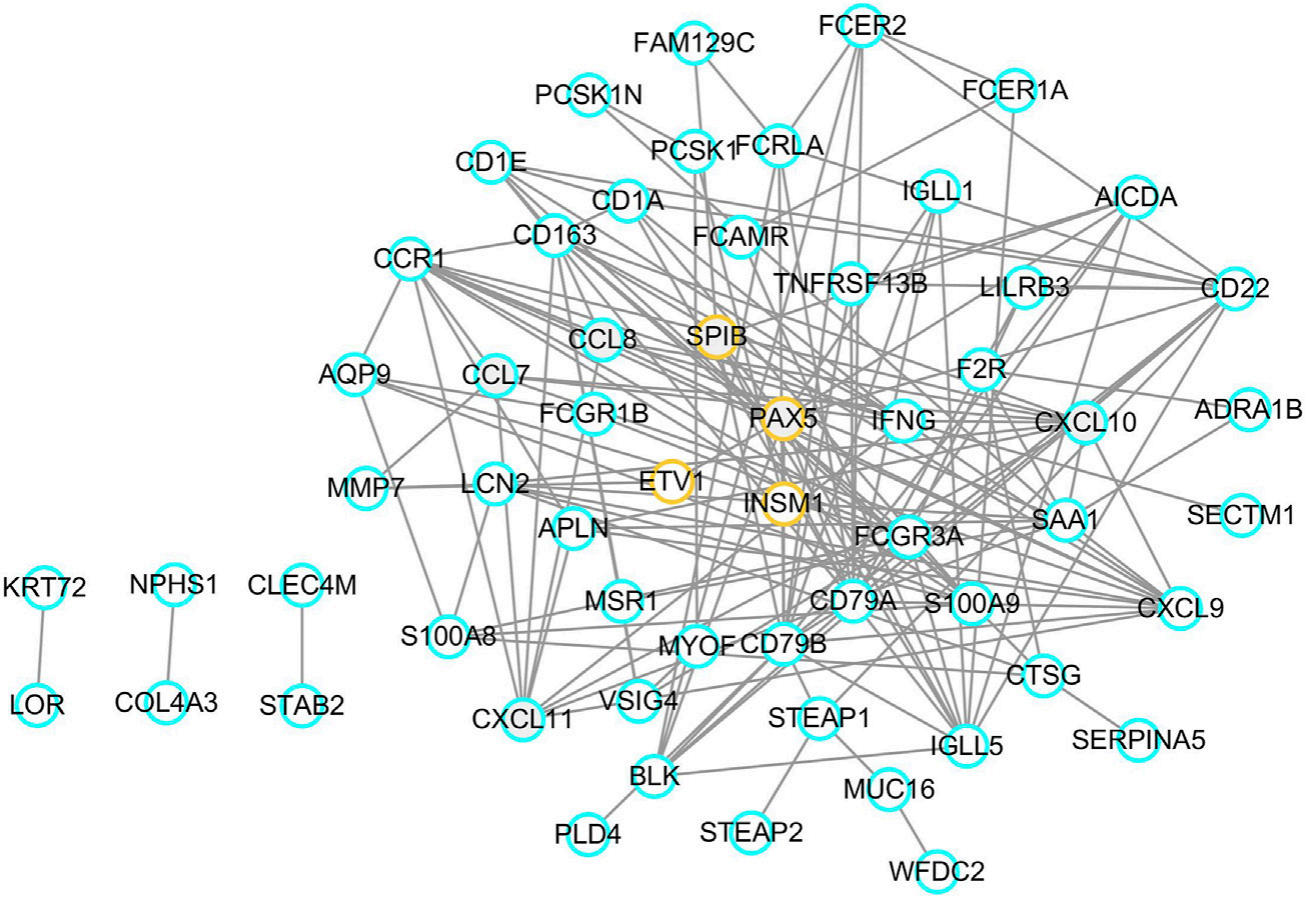
Transcription factor regulatory network. Orange circles represent transcription factors and blue circles mean common genes.

A list of 96 DEGs were uploaded to the STRING online database to obtain the PPI information. Afterwards, a total of 58 proteins and 150 edges were selected in the PPI network. These proteins were chosen based on a combined score ≥ 0.4 in STRING analysis. Next, four transcription factors were identified among the network nodes, including SPIB, PAX5, ETV1, and INSM1. Subsequently, a transcription factor regulatory network was built with the transcription factors and related hub proteins. As shown in Figure 5, orange circles indicate transcription factors, and blue circles indicate other common hub genes. These transcription factors interact with other common genes in a complex regulatory network involved in EBV positive PTCL-Us.

### Competing endogenous RNAs regulation network

**FIGURE 6:**
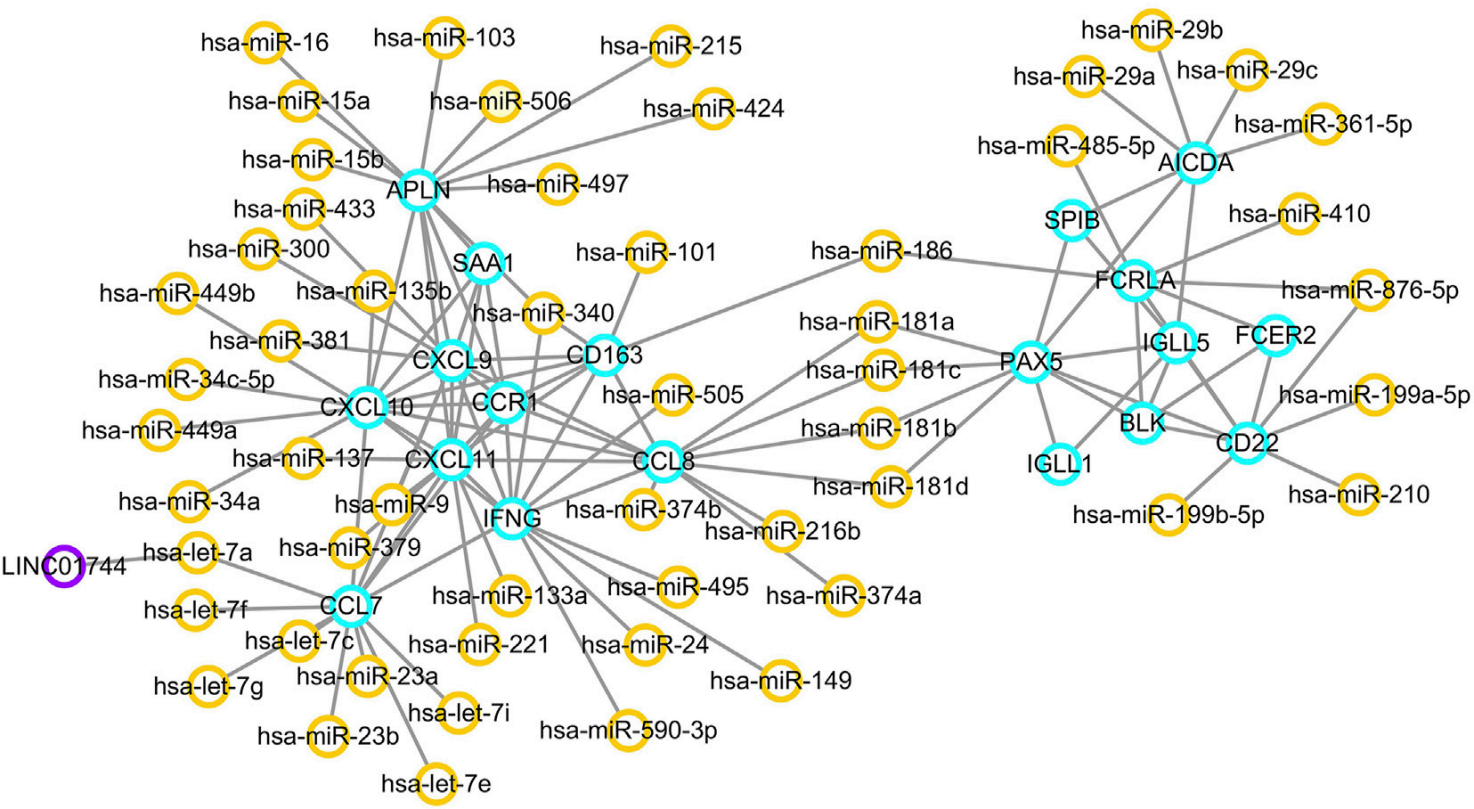
ceRNA regulatory network. Orange circles reflect miRNAs, and purple circles represent lncRNAs. Target genes are labeled in blue circles.

Using the MCODE package, two significant network modules were obtained from the PPI network. Afterwards, a systematic analysis on the interactions between miRNAs and the module proteins was carried out using TargetScan, microRNA.org and miRDB based on three different target prediction algorithms. The miRNA-mRNA regulatory pairs that were identified by all the three databases were used. As a result, a total of 54 miRNAs were found to be related to the identified modules. In particular, miR-186, miR-181a, miR-181b, miR-181c, and miR-181d were associated with both the two modules. The miRNA–targeted lncRNA interactions were predicted using starBase and Cytoscape software to construct a ceRNA interaction network. As a result, LINC01744 was identified to be highly involved in the module miRNA-mRNA interactions. After screening and identifying overlaps in the TargetScan, microRNA.org, miRDB and starBase databases, an integrated lncRNA–miRNA–mRNA network was established (Figure 6). In the ceRNA network, a total of 19 genes were regulated by 54 miRNAs and one LncRNA.

### Epstein-Barr virus related microRNAs regulation network

**FIGURE 7:**
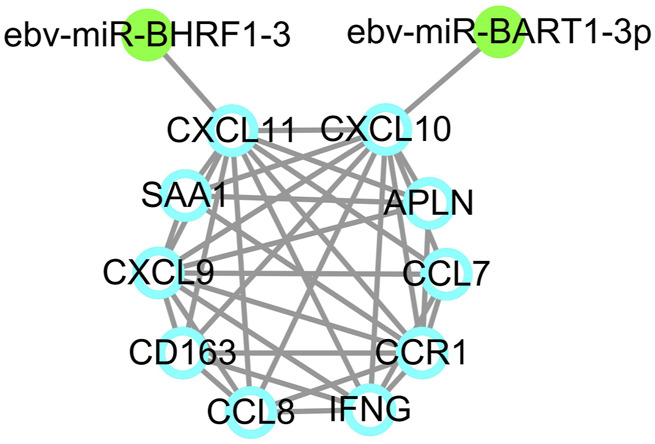
EBV-related miRNA regulatory network. EBV-related miRNAs are labeled in green dots while target genes are indicated by blue circles.

Since EBV positive PTCL-U is a very rare but aggressive disease entity, an EBV-related miRNA regulatory network was also studied. By predicting the EBV-related miRNAs of hub genes using ViRBase, we found that CXCL10 and CXCL11 in the most significant module were regulated by EBV-related miRNAs. In detail, CXCL10 was regulated by ebv-miR-BART1-3p, while CXCL11 was regulated by ebv-miR-BHRF1-3 (Figure 7).

### Network biomarkers in Epstein-Barr virus positive peripheral T cell lymphoma unspecified type

**FIGURE 8:**
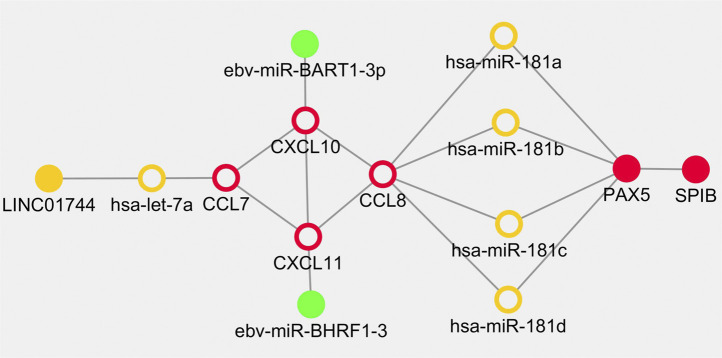
Dynamical network biomarkers for regulation in Epstein-Barr virus (EBV) positive peripheral T cell lymphoma unspecified type (PTCL-U). Target genes are labeled in red circles, transcription factors are reflected by red dots, miRNAs are indicated by orange circle, lncRNAs are represented in orange dots, and EBV-related miRNAs are illustrated in green dots.

Based on the above results, the overlapped genes and regulators including CXCL10, CXCL11, hsa-let-7a-5p, CCL7, CCL8, miR-181a/b/c/d, LINC01744, PAX5, SPIB, EBV-miR-BART1-3p and EBV-miR-BHRF1-3 were retrieved in the cross network. The regulatory pairs were integrated into a whole framework and identified as network biomarkers that may be highly associated with the initiation and progression of PTCL-U (Figure 8).

## Discussion

PTCL is an aggressive group of tumors characterized by heterogeneous morphological changes of mature T cell immunophenotypes (Pizzi et al., 2018). PTCL-U, the most common form of PTCL, is associated with poor prognosis (Tang et al., 2021). EBV infection has been increasingly emphasized as a poor prognostic indicator of PTCL-U and other lymphomas (Dupuis et al., 2006). EBV positive PTCL-U is rare but always associated with an aggressive clinical course (Dupuis et al., 2006). Previous studies have shown that the occurrence and development of cancer may be related to a dysregulated network of mRNAs, TFs, miRNAs, and lncRNAs (Bracken et al., 2016). In this study, we analyzed data from multiple databases that included mRNAs, TFs, miRNAs, and lncRNAs, and integrated them in a comprehensive regulatory network. These key network regulators may provide systematic insights into the mechanisms underlying the EBV positive PTCL-U.

Firstly, a total of 96 DEGs between PTCL-Us and normal controls were identified from the GEO database. Then, functional enrichment analysis was performed to explore the potential biological features of the identified DEGs. GO analysis showed that these genes were mainly associated with innate immune response, immune response, chemotaxis, chemokine-mediated signaling pathway, cell-cell signaling, B cell receptor signaling pathway, adaptive immune response at the BP level, significantly relevant to core cell structural at CC level and highly linked with the function of key molecules binding at the MF level including immunoglobulin receptor binding and CXCR3 chemokine receptor binding. Then, KEGG pathway enrichment analysis also illustrated that the DEGs were mainly involved in cytokine-cytokine receptor interactions, chemokine signaling pathways, hematopoietic cell lineage, primary immunodeficiency and intestinal immune network for IgA production. The GO and KEGG enrichment analysis that the DEGs were mainly related to the immune-related biological processes and regulation of some key molecules in immune response including chemokines, immunoglobulin receptors, and cytokines. As known to all, immunotherapy has been regarded as a powerful therapeutic strategy for various malignancies, including lymphoma. Failures of lymphoma immunotherapy may come from these mechanisms containing loss of major histocompatibility complexes, expression of immunosuppressive ligands, secretion of immunosuppressive cytokines, and the recruitment, expansion, and skewing of suppressive cell populations (Csizmar and Ansell, 2021). Previous studies have demonstrated that chemotactic responses play an important role in EBV-positive lymphoma processes, such as tumor vasculature development, cancer metastases and apoptosis (Wu et al., 2017). Studies have also shown that the cytokine-cytokine-receptor interaction pathway is correlated with the occurrence and development of acute T cell lymphoma, in which NOTCH1 and IKZF3 mutations play an important role (Mirji et al., 2016). The functional enrichment analysis results revealed that these DEGs may participate in the initiation and progression of EBV positive PTCL-U through affecting the immune system.

TFs function as key determinants in intrinsic cellular processes, including differentiation and development, and in the cellular response to external perturbation through specific signaling pathways (Weidemuller et al., 2021). TFs serve as a perfect bridge between the fields of gene regulation and signaling. We identified key TFs based on gene regulatory network of DEGs in EBV positive PTCL-U, including SPIB, PAX5, ETV1, and INSM1. SPIB is a member of the E-twenty-six (ETS) transcription factor family involved in regulating B cell development and differentiation (Xu et al., 2019) Moreover, previous studies have convinced SPIB as a novel prognostic biomarker in diffuse large B cell lymphoma that mediates apoptosis through the PI3K-AKT pathway (Takagi et al., 2016). PAX5 transcription factor, also known as B cell specific activator protein (BSAP), plays an important part in the hematopoietic system. Specific expression of PAX5 in B cells may serve as a biomarker in the diagnosis and prognosis of B cell leukemias and lymphomas (Shahjahani et al., 2015). ETV1 is an oncogenic transcription factor and is reportedly oncogenic and metastatic in several types of human cancers (Bordignon et al., 2019; Eid and Abdel-Rehim, 2019). Accumulating new evidence has identified that INSM1, a transcriptional regulator with a zinc-finger DNA-binding domain, may function as a cytoplasmic biomarker for neuroendocrine differentiation of tumor cells (Rosenbaum et al., 2015). These TFs may individually or collectively participate in the initiation and progression of EBV positive PTCL-U through regulating their target genes.

It is well established that ncRNAs play a crucial role in normal development of the hemolymph system and oncogenesis by regulating gene expression (Goodall and Wickramasinghe, 2021). Moreover, long ncRNAs, miRNAs, mRNAs may form a ceRNA network that plays an essential role in cancer pathogenesis (Yang et al., 2021). By building a ceRNA regulatory network, several key miRNAs and lncRNAs possibly involved in gene regulation associated with EBV positive PTCL-U were identified, including miR-181a/b/c/d, hsa-let-7a-5p, and LINC01744. The miR-181 family consists of miR-181a, miR-181b, miR-181c, and miR-181d that are specifically expressed in hematopoietic cells and control T cell repertoire selection through regulating multiple signaling pathways (Grewers and Krueger, 2020; Indrieri et al., 2020; Kim et al., 2021). Moreover, miR-181 family members, as the multifunctional miRNAs, play important part in tumorigenesis and cancer development, which may provide diagnostic and prognostic biomarkers or therapeutic targets in human cancer gene therapy (Rezaei et al., 2020). In addition, altered expression of miR-181 may affect cell fate and target drug resistance-associated mechanisms (Braicu et al., 2019). Previous studies have revealed that miR-181a-5p inhibits cancer cell migration and angiogenesis by down-regulating matrix metalloproteinase-14 (Li et al., 2015b). Studies have also identified miR-181b as a key regulator of the oncogenic process and indicated its clinical implications in cancer as a predictive and prognostic biomarker (Liu et al., 2014). Emerging evidence has supported that miR-181 also plays a crucial role in posttranscriptional regulation in the human acute T cell leukemia cell line and acute myeloid leukemia (Weng et al., 2015; Baghbani et al., 2018; Lee et al., 2019). Let-7a inhibits T cell proliferation by repressing STAT3, leading to the development of cell-based diseases (Hu et al., 2017). Besides, let-7a could regulate angiogenesis through post-transcriptional regulation of TGFBR3 (Wang et al., 2019b). Recent evidence revealed that EBV EBNA1 protein plays multiple roles in EBV latent infection and exerts their regulatory effects on viral latency through mediating let-7 miRNA and dicer (Mansouri et al., 2014). LncRNA LINC01744 was identified in the network; however, its relationship with EBV positive PTCL-U is still unknown, and more research is needed to elucidate it.

In addition, miRNAs could not only be produced by metazoans, but also by viruses, which may offer new insights into further research. To date, 44 EBV-encoded miRNAs have been found, most of which have been confirmed to exerts their regulatory effects in immune regulation and cancer initiation and progression by targeting host genes (Cai et al., 2006). EBV related miRNAs could desensitize cells to B cell receptor stimuli, and attenuate the downstream activation of NF-kappaB or AP1-dependent transcription (Chen et al., 2019). Most importantly, EBV-encoded miRNAs can exert immune escape function by targeting TAP2 protein in B and T cells to ultimately promote tumorigenesis (Hassani and Khan, 2019). Based on EBV related miRNA regulation network, we found that CXCL10 and CXCL11 were regulated by EBV-miR-BART1-3p and EBV-miR-BHRF1-3. EBV-miR-BART1 has been previously observed to be involved in tumor metastasis in nasopharyngeal carcinoma by regulating the target gene PTEN, and it also plays a role in EBV-associated gastric cancer (Cai et al., 2015; Jing et al., 2018). Moreover, EBV-miR-BHRF1-3 plays a role in the pathogenesis of multiple sclerosis (MS) and may be used as a diagnostic marker for prospective study about the pathogenesis of MS (Wang et al., 2017). As for the genes regulated by EBV-miRNAs, the CXCL9, CXCL10, CXCL11/CXCR3 axis has been found to regulate immune cell migration, differentiation, leading to tumor suppression and may have potential role in cancer treatment (Tokunaga et al., 2018). CXCL10 has been recognized as a strong angiostatic factors, and it may participate in the recruitment of tumor-infiltrating T cells (Karin and Razon, 2018). CXCL11 has various functions in the tumor microenvironment, such as suppressing angiogenesis, affecting cell proliferation, stimulating fibroblast directed carcinoma invasion, enhancing adhesion properties, inhibiting M2 macrophage polarization, and promoting the migration of certain immune cells (Gao and Zhang, 2021). Aberrant expression of these key genes may affect the infection status of EBV positive PTCL-U cases.

Cancers are highly complex and heterogeneous diseases. Thus, single biomarker is hard to uncover their evolutionary process. According to the preliminary study, combination biomarkers based on regulatory network may be more reliable with greater power for explaining the internal mechanisms (Peng et al., 2017a; Peng et al., 2017b; Peng et al., 2018). EBV positive PTCL-U is a rare and highly heterogeneous disease. Traditional research methods are difficult to deeply explore the underlying mechanisms. Bioinformatics and network analysis of gene microarrays are effective approaches to explore potential mechanisms in the pathogenesis of rare disease (Wasserman and Sandelin, 2004). In the current study, an integrated network module including some key regulators including TFs, miRNAs and lncRNAs involved in EBV positive PTCL-U was developed by combining the TF–mRNA, lncRNA–miRNA–mRNA, and EBV encoded miRNA–mRNA regulatory networks. In the cross network, a list of overlapping genes and regulators were observed. Interestingly, the shape of the cross network was very symmetric. First, CCL7, CCL8, CXCL10, and CXCL11 formed the core frame of the network, and they could be regulated by miR-181a/b/c/d, hsa-let-7a-5p, EBV-miR-BART1-3p, and EBV-miR-BHRF1-3, separately. In addition, hsa-let-7a-5p was also regulated by LINC01744. The transcription factor PAX5 could be regulated by miR-181(a/b/c/d) simultaneously, and PAX5 also has strong interactions with the transcription factor SPIB. The network-based approach of the present study identified potential biomarkers involved in EBV positive PTCL-U and provided a systemic method to integrate diverse information into a systematical framework.

Nevertheless, limitations exist in the present study. First, we failed used a similar number of cases of PTCL EBV-negative. This group as a control will be a more sound comparison, since comparing a EBV(+) PTCL with normal/reactive tissue is not going to reveal the significance of EBV but that of the neoplastic process itself. Second, the sample size for the rare disease is too small while investigating a larger sample size in EBV positive PTCL-U would help to confirm our data. Third, the data in the current study were identified by bioinformatics analysis, and the findings are warranted to be validated by further investigations. Thus, further biological experiments are needed to explore the biomarker value of the identified network regulators and the underlying mechanisms. Last, we should also pay attention to the quality control of the assay and data generation letter analysis process, and the combination of NGS, GEP, CNA and other multi-omics analysis, which is beneficial to the molecular typing of PTCL-U and the search for new therapeutic targets.

## Conclusion

In summary, a network-based approach to identify potential biomarkers involved in EBV positive PTCL-U was developed by integrating the regulatory networks, including TF-mRNA, lncRNA-miRNA-mRNA, EBV-encoded miRNA-mRNA. Two transcription factors, including PAX5 and SPIB, were identified that were both associated with the tumorigenesis of EBV positive PTCL-U through regulating their target genes. Four hub mRNAs in this network, including CXCL10, CXCL11, CCL7, and CCL8, were identified which have also been demonstrated to influence the tumor microenvironment and infection status. Four miRNAs (miR-181a, miR-181b, miR-181c, and miR-181d) and one unreported lncRNA, LINC01744, were identified in the ceRNA regulation network. In addition, EBV related miRNAs EBV-miR-BART1-3p and EBV-miR-BHRF1-3 were found to regulate CXCL10 and CXCL11 in the network. This study may provide a new avenue for investigating the regulatory mechanisms of EBV positive PTCL-U, and further molecular experiments are required to confirm these findings.

## Data Availability

The datasets presented in this study can be found in online repositories. The names of the repository/repositories and accession number(s) can be found below: NCBI GEO, accession no: GSE34143.

## Abbreviations

PTCL-U, peripheral T cell lymphoma unspecified type; EBV, Epstein-Barr virus; TFs, transcription factors; DEGs, differentially expressed genes; GO, Gene Ontology; KEGG, Kyoto Encyclopedia of Genes and Genomes; PPI, protein–protein interaction; DAVID, Database for Annotation, Visualization, and Integrated Discovery; BP, Biological processes; CC, Cell component; MF, Molecular function.
